# Nanozinc and plant growth-promoting bacteria improve biochemical and metabolic attributes of maize in tropical Cerrado

**DOI:** 10.3389/fpls.2022.1046642

**Published:** 2023-01-12

**Authors:** Arshad Jalal, Carlos Eduardo da Silva Oliveira, Andréa de Castro Bastos, Guilherme Carlos Fernandes, Bruno Horschut de Lima, Enes Furlani Junior, Pedro Henrique Gomes de Carvalho, Fernando Shintate Galindo, Isabela Martins Bueno Gato, Marcelo Carvalho Minhoto Teixeira Filho

**Affiliations:** ^1^ Department of Plant Protection, Rural Engineering and Soils (DEFERS), São Paulo State University (UNESP), Ilha Solteira, Brazil; ^2^ Department of Plant Science, Food Technology and Socio-Economics, São Paulo State University (UNESP), Ilha Solteira, Brazil; ^3^ Center for Nuclear Energy in Agriculture (CENA), University of São Paulo (USP), Piracicaba, Brazil

**Keywords:** PGPB (plant growth-promoting bacteria), photosynthesis, plant growth, nutrient uptake, storage proteins, amino acids, zinc fertilization, grain yield

## Abstract

**Introduction:**

Plant growth-promoting bacteria (PGPBs) could be developed as a sustainable strategy to promote plant growth and yield to feed the ever-growing global population with nutritious food. Foliar application of nano-zinc oxide (ZnO) is an environmentally safe strategy that alleviates zinc (Zn) malnutrition by improving biochemical attributes and storage proteins of grain.

**Methods:**

In this context, the current study aimed to investigate the combined effect of seed inoculation with PGPBs and foliar nano-ZnO application on the growth, biochemical attributes, nutrient metabolism, and yield of maize in the tropical savannah of Brazil. The treatments consisted of four PGPB inoculations [i.e., without inoculation*, Azospirillum brasilense* (*A. brasilense*)*, Bacillus subtilis* (*B. subtilis*)*, Pseudomonas fluorescens* (*P. fluorescens*), which was applied on the seeds] and two doses of Zn (i.e., 0 and 3 kg ha^−1^, applied from nano-ZnO in two splits on the leaf).

**Results:**

Inoculation of *B. subtilis* with foliar ZnO application increased shoot dry matter (7.3 and 9.8%) and grain yield (17.1 and 16.7%) in 2019-20 and 2020-2021 crop seasons respectively. Inoculation with A. brasilense increased 100-grains weight by 9.5% in both crop seasons. Shoot Zn accumulation was improved by 30 and 51% with inoculation of P. fluorescens in 2019-20 and 2020-2021 crop seasons. Whereas grain Zn accumulation was improved by 49 and 50.7% with inoculation of *B. subtilis* and P. fluorescens respectively. In addition, biochemical attributes (chlorophyll a, b and total, carotenoids, total soluble sugar and amino acids) were improved with inoculation of *B. subtilis* along with foliar nano ZnO application as compared to other treatments. Co-application of P. fluorescens with foliar ZnO improved concentration of grains albumin (20 and 13%) and globulin (39 and 30%). Also, co-application of *B. subtilis* and foliar ZnO improved concentration of grains glutelin (8.8 and 8.7%) and prolamin (15 and 21%) in first and second seasons.

**Discussion:**

Therefore, inoculation of *B. subtilis* and *P. fluorescens* with foliar nano-ZnO application is considered a sustainable and environmentally safe strategy for improving the biochemical, metabolic, nutritional, and productivity attributes of maize in tropical Savannah regions.

## 1 Introduction

Environmental disaster, food, and nutritional insecurities are the foremost devastating challenges to the agricultural sector. Malnutrition is a global dietary concern and one of the most a serious threats to agriculture crop production systems, affecting over half of the global population (Ramzan et al., 2020). Zinc (Zn) is one of the key dietary nutrients and its malnutrition has affected over one-third of agricultural soils because of the presence of excessive soil carbonates, oxides, silicates, and phosphates, as well as through extensive farming systems and practices (Masood et al., 2022). Zn is an essential micronutrient for the normal growth, development, and physiological activities of each living organism (Stanton et al., 2022). In addition, Zn is involved in numerous metabolic and biochemical functions of plants, such as protein and chlorophyll synthesis, lipid and carbohydrate metabolism, enzymatic activities and photosystems, pollen fertility, and energy production (Suganya et al., 2020; Zafar et al., 2022). Zn is responsible for the stabilization and catalyzation of ≈10% of human body proteins, and it presence helps in the mitigation of reactive oxygen species (ROS) through antioxidant metabolism and lipid peroxidation of cell membranes (Ojeda-Barrios et al., 2021; Li et al., 2022). Plants are the major source of Zn entrance into human body. Therefore, a quick and inexpensive alternative strategy is needed to improve Zn bioavailability in edible tissues and crop productivity to combat malnutrition and food security.

Nanotechnology is an ecofriendly alternative that increases targeted nutrient concentration and metabolism, as well as photosynthetic machinery of the chosen crop (Kapoor et al., 2022). Nano-fertilizer with zinc oxide (ZnO) is being recognized as an important and effective alternative for increasing growth and productivity by regulating primary photosynthetic activities and carbohydrate metabolism to satisfy the nutritional quality of plants (Singh et al., 2021; Jalal et al., 2022c). Nano-fertilizer reduces the use of synthetic fertilizers while increasing targeted nutrient availability for plant uptake and its intake by human in edible grains (Prasad et al., 2017). Foliar application of nano-fertilizer has been widely reported for enhancing plant nutrition and productivity, as it enters the cell membrane more effectively, contributing to the metabolism of proteins, sugars, and amino acids, and photosynthesis of plants to increase nutrient use efficiency and reduce environmental constraints (Weisany et al., 2021; Kandil et al., 2022). Foliar spray of ZnO is a more viable and prompt strategy than root/soil Zn application because of the large surface area and direct absorption through stomata and cuticles, and then translocation *via* the phloem into the chloroplast (Su et al., 2019; Zhu et al., 2020). The delivery of nano-Zn enhances plant growth, productivity, and Zn concentration in the edible tissues (Dimkpa et al., 2022). However, these benefits are still to be adapted at field scale because of the nature and size of particulates (Sudhakaran et al., 2020). Hence, the introduction of plant growth-promoting bacteria (PGPBs) in combination with nano-Zn fertilizer could be a better integrated alternative to improve agricultural productivity in a more sustainable and ecofriendly way to the environment.

PGPBs are applied *via* seeds, soil, and leaves to enhance efficiency of plant growth and manage abiotic stresses through root morphological alterations (Goswami and Suresh, 2020). Seed inoculation with PGPBs is a promising strategy to promote plant growth and development by facilitating nutrient use efficiency, modulating hormonal activities, and inhibiting pathogenic infestation (Di Benedetto et al., 2017; Kumar et al., 2019). In addition, PGPBs contribute to the synthesis of secondary metabolites, water absorption, nutrient [phosphorus (P), Zn, and potassium (K)] solubilization, and tolerance to biotic and abiotic stresses (Hungria et al., 2018; Jalal et al., 2021; Lopes et al., 2021). The inoculants of the genus *Azospirillum* are being recognized in the biosynthesis of auxin synthesis, nutrient cycling and availability, and biological nitrogen (N) fixation by reducing N_2_ into ammonia (NH_3_) (Bhat et al., 2019; Carrillo-Flores et al., 2022; Galindo et al., 2022). *Bacillus subtilis* (*B. subtilis*) has the ability to promote plant growth through P solubilization, increase Zn use efficiency, bioremediation of heavy metals, and controlling phytopathogenic infestation, which in turn leads to increased root–shoot development and productivity (Lobo et al., 2019; dos Santos et al., 2021; Jalal et al., 2022a). In addition, *Pseudomonas fluorescens* (*P. fluorescens*) is considered to be one the most effective inoculants to synthesis antibiotics, metabolites, and volatile organic compounds to combat soil pathogens (David et al., 2018), improving Zn and P concentrations (Jalal et al., 2022b; Rosa et al., 2022), and also helping in N-fixing activities for sustainable crop production (Jing et al., 2020; Agbodjato et al., 2021).

PGPBs could increase Zn solubility and uptake through the production of organic and inorganic acids, and several chelators (Idayu et al., 2017; Khoshru et al., 2020). Green-synthesized ZnO increases morphological and biochemical attributes that lead to sustainable crop production systems (Natarajan et al., 2021). Zinc fertilization in combination with inoculation of *Azospirillum brasilense* (*A. brasilense*) increases Zn use efficiency and accumulation, and yield of cereal crops grown in tropical environments (Galindo et al., 2021). In addition, *B. subtilis* and *P. fluorescens* are being recognized as the most effective inoculants to solubilize Zn and P, and improve plant growth and development under different climatic conditions (Rosa et al., 2020; Ahmad et al., 2021; 2022b; Jalal et al., 2021; Jalal et al., 2022a).

Maize is recognized as the “queen of cereals” because of its extensive use and flexibility. It is the most frequently cultivated grain crop, serving as a major source of nutrition in many developing countries (Kumawat et al., 2020). Therefore, it is important to adapt new biotechnology, like the use of nano-fertilizers and PGPB inoculation, for improving physiochemical and yield traits of maize under changing environmental conditions. The literature is lacking data on the combined effects of PGPBs and nano-Zn on growth and development, and nutritional status of maize in the tropical savannah of Brazil. There exists a research gap on the effect of PGPBs and nano-Zn on primary metabolic and biochemical attributes, and yield of maize crop in the tropical savannah of Brazil. In this context, it was hypothesized that inoculation with PGPBs and foliar nano-Zn fertilization would be an interesting strategy to improve primary metabolic and biochemical attributes, and the yield of the maize crop. Therefore, the objective of the study was to evaluate the effect of inoculation with PGPBs in association with or without foliar nano-Zn application on the levels of chlorophyll a, b, and total chlorophyll, and concentrations of amino acids, sucrose, and total sugar in maize. In addition, we wanted to know the effect of PGPBs and foliar nano-Zn spray on the uptake of Zn in shoot and grains, and the grain yield of maize in the tropical savannah of Brazil.

## 2 Materials and methods

### 2.1 Description of experimental site

**Figure 1:**
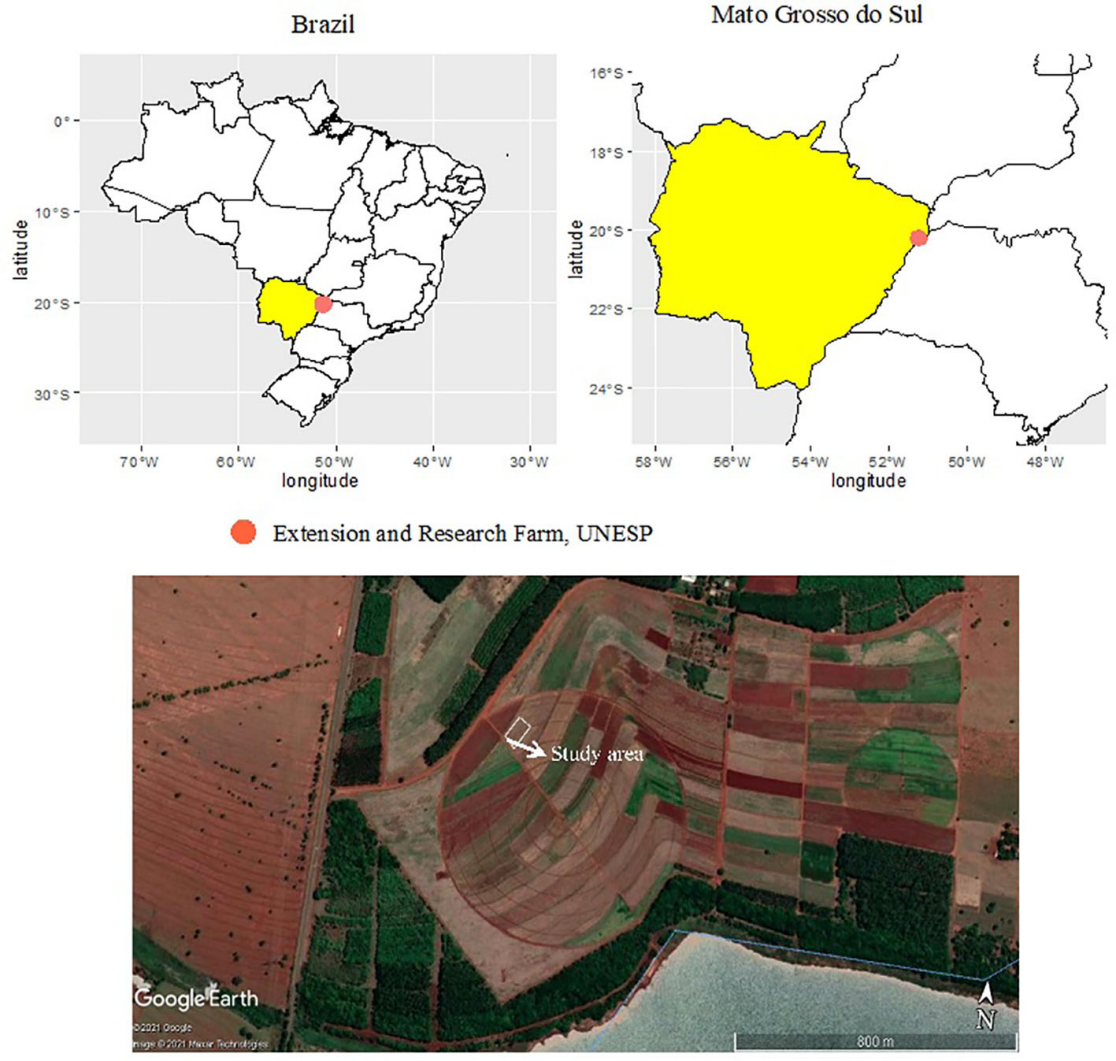
Geographical location of experimental site at Extension and Research Farm, UNESP–Ilha Solteira, at Selvíria, state of Mato Grosso do Sul, Brazil (20°22′ S, 51°22′ W, altitude of 335 m) during the 2019–20 and 2020–1 cropping seasons. The map was created using pacot, geobr, and ggplot within R software (R Core Team, 2015). Accessed on 27 February 2022. Projection System WGS 84/UTM 200DC [EPSG: 4326]. This image was taken from the Google Earth program, Google Company (2021). Map data: Google, Maxar Technologies.

Two field experiments with maize were performed during the summer (October–March) of 2019–20 and 2020–1 cropping seasons at the Extension and Research Farm of School of Engineering, São Paulo State University (UNESP) at Selvíria, Mato Grosso do Sul, Brazil. The site is located at geographical coordinates of 20°22′ S latitude, 51°22′ W longitude, and an altitude of 335 m (**Figure 1**).

The soil is clayey oxisol defined as Rhodic Haplustox (Soil Survey Staff, 2014) and Red Latosol Dystrophic (Santos et al., 2018), with a granulometric characterization of 777, 98, 125 g kg^-1^ of sand, silt and clay at a soil depth of 0.00–0.25 m (Teixeira et al., 2017). The experimental site has a history of more than 30 years’ cultivation with an annual cereal–legume crop rotation. In addition, the site was under a no-tillage system for the last 13 years while wheat was cultivated prior to the current maize experiments in both years.

**Figure 2:**
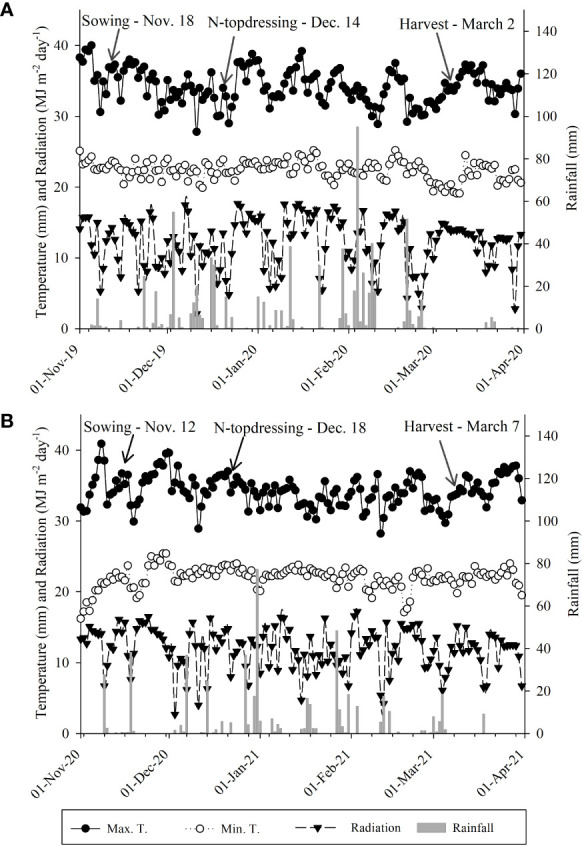
Rainfall, maximum and minimum temperatures, and light radiation during the experimental period at a weather station of the Extension and Research Farm of School of Engineering– UNESP, November to March 2019–20 **(A)** and 2020–21 **(B)**, respectively.

The experimental region is characterized as Aw-Köppen with a rainy summer (an average rainfall of 1370 mm and 23.5°C), and is humid and tropical with a relative humidity of 70–80% (Alvares et al., 2013). Different climatic factors (e.g., rainfall, temperature, and light radiation) during the current experiments, in both cropping seasons, were carefully monitored (**Figure 2**).

### 2.2 Soil analysis

Twenty random soil samples were collected before the experiment started from a soil layer of 0.00–0.20 m in both cropping seasons. The collected samples were properly mixed to attain a composite sample, then air-dried, sieved (2 mm), and prepared for chemical characterization (Raij et al., 2001). The soil chemical characterization is summarized in **Table 1**.

**Table 1:** Pre-maize experiment soil analysis of composite sample in a soil layer (0–0.20 m) in the 2019–20 and 2020–1 cropping seasons.

Property	Unit	Status	
		2019–20	2020–1
pH (CaCl_2_)	**—**	5.2	5.3
Organic matter	mg dm^-3^	18	23
P (resin)	mg dm^-3^	38	40
K	mmol_c_ dm^-3^	1.7	1.9
Ca	mmol_c_ dm^-3^	21	22
Mg	mmol_c_ dm^-3^	15	12
B (hot water)	mg dm^-3^	0.14	0.39
Cu (DTPA)*	mg dm^-3^	3.4	3.7
Fe (DTPA)*	mg dm^-3^	25	28
Zn (DTPA)*	mg dm^-3^	0.7	9.4
Mn (DTPA)*	mg dm^-3^	38.1	37.3
S-SO_4_	mg dm^-3^	4.0	22
H+Al	mmol_c_ dm^-3^	34	31
CEC (pH7)*	mmol_c_ dm^-3^	75.7	66.9
V*	%	50	54

*CEC, cation exchange capacity; V, base saturation; DTPA, diethylenetriaminepentaacetic acid.

### 2.3 Experimental design and treatments

The experiments were conducted in a randomized complete block design, with four replications in 4 × 2 factorial scheme. There were four types of seed inoculation with PGPBs (i.e., no inoculation, *A. brasilense, B. subtilis*, and *P. fluorescens*) and two foliar nano-ZnO applications (i.e., without or with 3 kg Zn ha^−1^), applied at 50% tasseling and at the grain setting/filling stage of maize.

The maize seeds were chemically treated with Standak Top^™^, a co-formulation of fungicide [arbendazim  +  thiram (45 g  +  105 g of active ingredient (a.i.) 100 kg^−1^ seeds] and insecticide [imidacloprid  +  thiodicarb (45 g  +  135 g of a.i. 100 kg^−1^ seeds)] 24 h prior to inoculation. Treating cereal seeds with Standak Top^™^ is a common practice in the Brazilian tropical savannah to prevent soil pathogen infection without any harmful effects on the bacterial inoculation (Munareto et al., 2018; Cardillo et al., 2019).

Seeds were manually inoculated by mixing seeds and the respective inoculant in a plastic bag 1 h before sowing. Inoculation with *A. brasilense* strains Ab-V5 (CNPSo 2083) and Ab-V6 (CNPSo 2084) was carried out at a dose of 200 ml of liquid inoculant per 24 kg of seeds with a guarantee of 2 × 10^8^ CFU ml^−1^, whereas *B. subtilis* strain (CCTB04) was inoculated with a guarantee of 1 × 10^8^ CFU ml^−1^ and *P. fluorescens* strain (CCTB03) with a guarantee of 2 × 10^8^ CFU ml^−1^, at a liquid inoculant dose of 150 ml ha^−1^ per 24 kg of seeds. The inoculation was carried out by following the recommendation of the inoculant-providing company (Biotrop^®^, Curitiba, Brazil). These inoculants are commercially used in Brazil with strains of *A. brasilense* (AzoTotal™), *B. subtilis* (Vult™), and *P. fluorescens* (Audax™) used to promote growth and productivity. The gene sequencing of *A. brasilense* highlighted that strains Ab-V5 and Ab-V6 are carrying *fix* and *nif* genes, which promotes nutrient cycling and availability, biological N fixation, auxin production, and induces plant tolerance against biotic and abiotic stresses (Fukami et al., 2017; Fukami et al., 2018b; Galindo et al., 2021). *B. subtilis* is the first Gram-positive bacterium carrying non-ribosomal peptide synthetases and beta-glucanase that are resistant to phytopathogen attack and facilitate heavy metal accumulation, while *zntR* as Zn transporter induces plant growth promotion (Chaoprasid et al., 2015; Rekha et al., 2017; Muñoz-Moreno et al., 2018). *P. fluorescens* is considered an efficient biocontrol agent, with the synthesis of antibiotics and volatile organic compounds to deter soil pathogens, and helping in gluconic acid production, solubilization of nutrients, and biological N fixation (David et al., 2018; Jing et al., 2020).

The foliar Zn application was performed from a liquid source of Zn (Nano R1 zinco^™^) that was obtained from Allplant^®^ fertilizers industry, São Paulo, Brazil. The company is already registered with the Ministry of Agriculture, Brazil. Nano R1 zinc is a fluid suspension with 50% p/p Zn, 1000 g/l solubility, and 2.0 density, and has been successfully used in previous studies (Nakao et al., 2018; Jalal et al., 2022c). A total of 3 kg ha^−1^ of ZnO was applied in two splits, 50% Zn at V8/V10 and 50% at R1 stage of maize (Stewart et al., 2020). The application was performed through a manual sprayer pump with a 6.0-l water capacity (300 l/ha of volume application). The field was inspected soon after the foliar spray and no leaf damage was observed.

### 2.4 Field management

The field site was sprayed with glyphosate (Roundup™) + 2,4-D (1800 + 670 g ha^−1^ of a.i.) 15 days prior to the experiment being planted to control pre-emerged weeds. A simple maize hybrid FS500PWU-Forseed (registered with the National Technical Commission on Biosafety of Brazil under reference n°. 1596/2008 for tropical and sub-tropical regions) was planted on 18 November 2019 and 12 November 2020 in a no-tillage system at 3.3 seeds m^−1^. All the treatments were uniformly fertilized with 350 kg ha^−1^ NPK (08 : 28 : 16, urea) on the basis of the soil analysis and expected yield. Seedlings emerged after 5 days of planting in both experimental years. Each experimental plot consisted of six 6-m-long maize rows with a 0.45m between rows. The total plot size was 16.2 m^2^. The data were collected from four central rows with a useful area of 10.8 m^2^. The post-emergence weeds were controlled by spraying herbicides atrazine and tembotrione (1000 + 84 g a.i. ha^−1^) at the V3 growth stage of maize. N side dressing (120 kg ha^−1^, applied in the form of ammonium sulphate; 21% N) at V6 growth stage (i.e., 30 and 31 days after emergence in the 2019–20 and 2020–1 maize cropping seasons, respectively) was applied to all treatments to uniformly distribute on the soil surface and was incorporated, by central pivot irrigation, on the same day. Irrigation was performed usinfg a central pivot sprinkler irrigation system at 14-mm water volume on a shift of 72 hours or as per crop requirement. The crop was manually harvested on 2 March 2020 and 7 March 2021.

### 2.5 Assessments and evaluations

#### 2.5.1 Growth and productivity attributes

Plant height was determined by measuring plant length from the surface of the ground to the upper apex of the tassel. The plants from four central lines were harvested, dried, and weighed with an analytical balance for the analysis of shoot dry matter. Ten random ears were collected at harvest to count number of rows and grains per ear. One-hundred-grain mass was measured with a precise scale at 13% humidity (wet basis). The ears from the central lines of each plot were manually harvested, threshed mechanically, and the grain weight was converted into kg ha^−1^ at 13% humidity to quantify yield.

#### 2.5.2 Zinc nutrition and use efficiency

Zn accumulation in shoot and grains was estimated from the ratio of Zn concentration in shoot and grains, and shoot dry matter and grain yield, respectively. Shoot and grain Zn concentrations were determined by nitroperchloric digestion and quantified with atomic absorption spectrophotometry, following the protocols of Malavolta et al. (1997). Zinc use efficiency (ZnUE), *via* Eq. 1, and applied Zn recovery (AZnR), *via* Eq. 2, were calculated according to the methodology of Fageria et al. (2011):


(1) $$ZnUE=\frac{GYF-GYC}{applied\;Zn\;dose}$$

(2) $$AZnR=\frac{GSZnAF-GSZnAC}{applied\;Zn\;dose}$$

Where GYF is the grain yield with nano-Zn foliar fertilization, GYC is the grain yield in the control treatments, GSZnAF is the grain plus shoot Zn accumulation in nano-Zn-applied treatments, and GSZnAC is grain plus shoot Zn accumulation in control treatments.

#### 2.5.3 Photosynthetic pigments

The photosynthetic pigments (chlorophyll a, b, and total, and carotenoid) were extracted and analyzed by the procedure of Lichtenthaler (1987). Fresh leaves were collected at flowering stage. The samples of 0.5 g were macerated in liquid nitrogen and 50 ml of 80% acetone, stored in the refrigerator and then centrifuged at 10,000 × *g* for 10 min. The absorbance of the acetone extracts were quantified at 663, 645, and 470 nm using a UV-160 A UV–vis spectrometer for chlorophyll a, total, and chlorophyll b and carotenoids concentrations, respectively.

#### 2.5.4 Primary metabolism assay

##### 2.5.4.1 Extraction for total soluble sugar and amino acids

Total soluble sugar (TSS) and amino acids were extracted from lyophilized leaves (≈0.5 g) in 10 ml of MCW solution (60% methanol, 25% chloroform, and 15% water) according to the procedure of Bileski and Turner (1966). The material solution was homogenized in a 15-ml polystyrene tube by vortexing, placed in a refrigerator for 48 h and centrifuged at 10,000 rpm for 10 min at 4°C. A 5-ml MCW extract supernatant was collected in a tube, and 1 ml of chloroform and 1.5 ml of distilled water added. After 24 h, the separation phase of aliquots from the hydrophilic portion was used for the determination of total soluble sugar and amino acid concentrations.

##### 2.5.4.2 Determination of total soluble sugar

TSS in maize leaves was quantified according to the procedure of Dubois et al. (1956). A 20-µl MCW extract was mixed with 500 µl of 5% phenol (w/v) and 2 ml of concentrated H_2_SO_4_ in a glass tube. After homogenization in a vortex mixer, the tube was heated at 100°C for 10 min and then cooled down to room temperature. Afterward, the readings were performed at an absorbance of 490 nm in spectrophotometer (SP-220, bioespectro™). The standard sucrose curve was used to quantify total sugar concentration and was expressed in mg g^−1^ fresh weight (FW).

##### 2.5.4.3 Determination of total amino acids

The protocols of Cocking and Yemm (1954) were used to quantify variation in total free amino acid concentration in maize leaves. An aliquot of 300 µl of MCW extract was mixed with 500 µl of 0.2 M sodium citrate, 200 µl of 5% ninhydrin in ethylene glycol, and 1 ml of 0.0002 M KCN solution in a glass tube. The content of the tubes was homogenized by vortexing and heated at 100°C for 20 min, and then cooled with tap water for ≈10 min. After cooling to room temperature, 1 ml of 60% ethanol was added to the glass tube and homogenized by vortexing. The readings were obtained at and absorbance of 570 nm using a spectrophotometer (SP-220, bioespectro™). The methionine standard curve was used to calculate free amino acid concentration and was expressed in mg g^−1^ FW.

##### 2.5.4.4 Determination of storage proteins

The concentration of grain storage proteins (e.g., albumin, globulin, prolamin, and glutelin) was determined according to the protocols of Bradford (1976). Dried and ground grain samples of 0.25 g was extracted with 5 ml of deionized water in 15-ml falcon tubes. The material was homogenized by vortexing for 1 min and then centrifuged at 10,000 rmp for 20 min at 4°C. A 20-μl supernatant was extracted with 1 ml of Bradford’s solution into 2-ml micro-tubes. The samples were homogenized, and read at an absorbance of 595 nm using a spectrophotometer (SP-220, bioespectro™) for the sequential extraction of albumin concentration. The same sample was used for the quantification of globulin by replacing water with 5 ml of 5% sodium chloride (NaCl) then replaced NaCl with 5 ml of 60% ethanol to determine prolamin concentration. Finally, the glutelin fraction was quantified with 5 ml of 0.4% sodium hydroxide. Bovine serum albumin was used as a standard and was expressed in mg g^−1^ dry mass.

### 2.6 Statistical analysis

The entire dataset was tested for normality using the Shapiro–Wilk test and Levene’s homoscedasticity test (*p*< 0.05), which showed that data were to be normally distributed (*W* ≥ 0.90). The data were subjected to analysis of variance (*F*-test) where foliar nano-Zn spray, PGPB inoculations, and their interactions were considered fixed variables, and replication was considered a random variable in the model. When a main effect or interaction was observed as being significant by *F*-test (*p* ≤ 0.05), then Tukey’s test (*p* ≤ 0.05) was used for mean comparison of nano-Zn spray and PGPB inoculation using R software (R Core Team, 2015).

A Pearson correlation analysis (*p* ≤ 0.05) was conducted, and a heatmap was created using corrplot package of “color” and “cor.mtest” functions to calculate coefficients and evaluate the relationships between growth, yield, nutritional, biochemical, and metabolic attributes of maize using R software (R Core Team, 2015).

A principal component analysis (PCA) was used to evaluate maize growth, grain yield and components, and nutritional, biochemical, and metabolic attributes in both years of study. The PCA was performed using factoextra and FactoMineR packages in R software (R Core Team, 2015). The number of principal components (PCs) was selected based on eigenvalues. The biplot graphs represent PC1 on the *x*-axis and PC2 on the *y*-axis of the plot.

## 3 Results

### 3.1 Growth, yield components, and yield of maize

**Figure 3:**
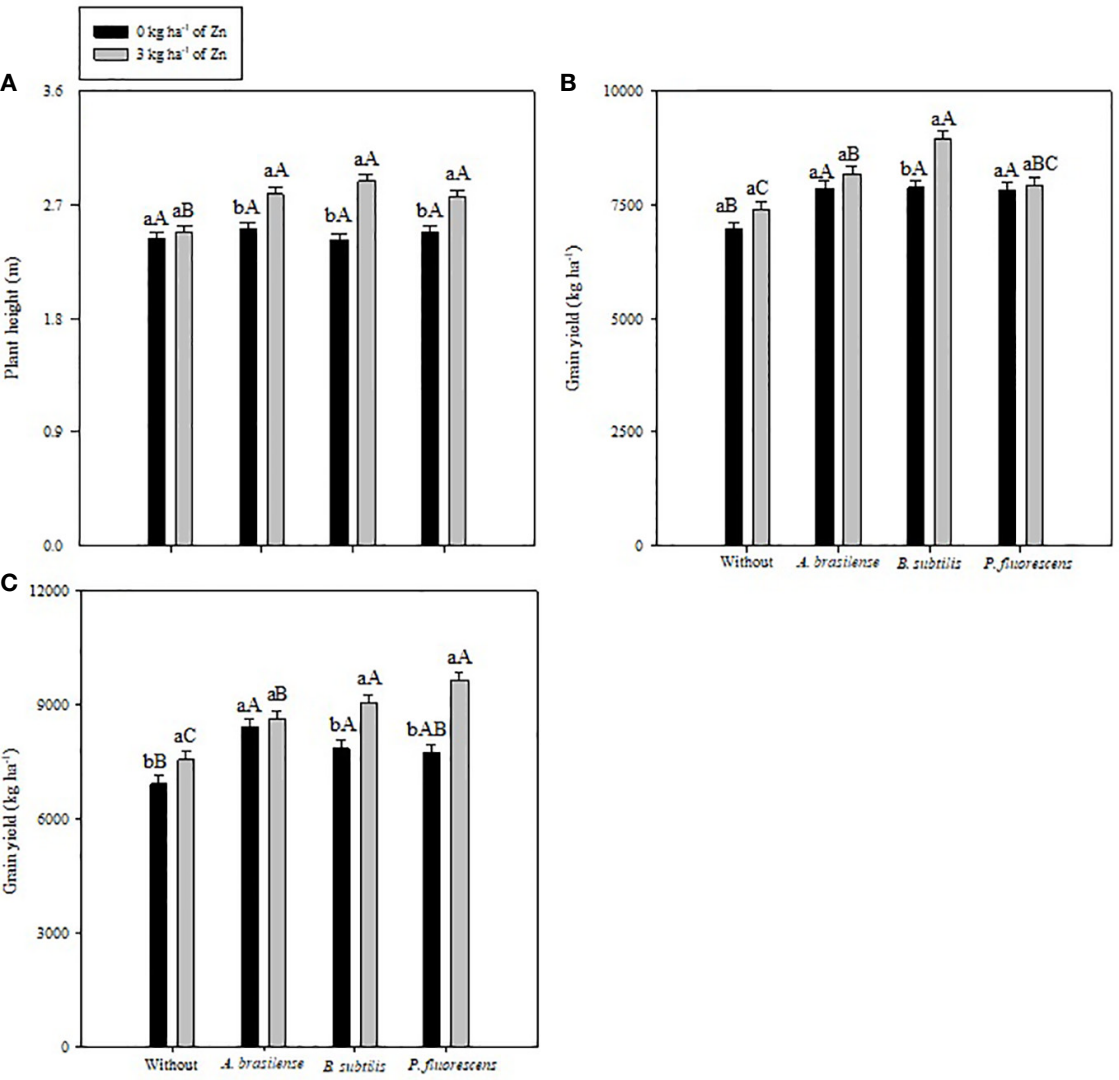
Plant height in 2020–1 **(A)**, and grain yield in 2019–20 **(B)** and 2020–1 **(C)** of common bean as a function of plant growth-promoting bacteria in combination with or without foliar nano-zinc oxide (ZnO) application. Without = control (no inoculation). The uppercase letters compare interactions of inoculations within each dose of foliar nano-ZnO application and lowercase letters are used to compare interactions of foliar zinc doses (presence and absence) within each inoculation treatment. The identical alphabetic letters do not differ from each other by Tukey’s test (*p*< 0.05) for foliar ZnO doses and inoculations in the 2019–20 and 2020–1 cropping seasons. Error bars indicate standard error of the mean (*n* = 4 replications).

The current study addressed the impact of PGPBs and foliar nano-Zn application on the growth performance and nutrient metabolism of a maize crop in a tropical savannah region. Inoculation with PGPBs increased plant height in both years of study, whereas foliar nano-Zn and interaction of foliar nano-Zn and PGPBs did not influence plant height in the 2020–1 maize cropping season (**Table 2**). The interaction of foliar nano-Zn and PGPBs for plant height in the 2019–20 cropping season was significant (**Figure 3A**). Foliar nano-Zn at a dose of 3 kg ha^−1^, along with inoculation of *A. brasilense*, *B. subtilis*, and *P. fluorescens* produced taller maize plants. All inoculation treatments were observed with taller plants under foliar nano-Zn application compared with the control treatments. There was no significant difference among inoculation treatments in the absence of foliar nano-Zn application (**Figure 3A**).

**Table 2:** Plant height, shoot dry matter, and number of rows per cob of maize as a function of plant growth-promoting bacteria inoculation, together with or without nano-zinc oxide spray in the 2019–20 and 2020–1 cropping seasons.

Treatment	Plant height			Shoot dry matter		Number of rows cob^-1^	
	——— m ———			—— kg ha^-1^ ——		———–	
	2019–20	2020–1		2019–20	2020–1	2019–20	2020–1
Inoculation (I)							
Without	2.45		2.75 a	11526 b	11359	16.93 b	16.03 b
*A. brasilense*	2.64		2.74 ab	12157 a	12196	17.62 ab	16.91 ab
*B. subtilis*	2.65		2.64 b	12368 a	12478	17.81 a	16.80 ab
*P. fluorescens*	2.62		2.74 ab	12119 a	12623	17.25 ab	17.17 a
Foliar zinc (ZnF) spray (kg ha^-1^)							
0	2.45		2.69	11595 b	11521 b	17.22	16.40 a
3	2.73		2.74	12490 a	12807 a	17.59	17.05 b
F-test							
I	6.6^*^		4.2^*^	9.6^**^	2.0^ns^	4.0^*^	3.8^*^
ZnF	54.6^**^		3.2^ns^	59.3^**^	10.6^**^	3.7^ns^	6.5^*^
I × ZnF	5.3^*^		2.0^ns^	1.7^ns^	0.13^ns^	0.5^ns^	0.24^ns^
**CV (%)**	3.9		2.6	2.7	9.2	3.2	4.3

Means in the column followed by different letters are statistically different by Tukey’s test, p ≤ 0.05. ^**^ and ^*^ significant at p< 0.01 and p< 0.05, respectively, while ^ns^ is non-significant by F-test.

The interaction of PGPBs and nano-Zn foliar spray was not significant for shoot dry matter of maize. However, the effect of foliar nano-Zn spray was significant in both cropping seasons (**Table 2**). Nano-Zn foliar spray increased shoot dry matter of maize by 7.7% and 11.2% in the 2019–20 and 2020–1 cropping seasons, respectively. In addition, plants inoculated with *B. subtilis* were observed as having greater shoot dry matter (12,368 kg ha^−1^), which was statistically at per with other inoculations, than the control treatment. There were no statistical differences among treatments regardless of inoculation in the 2020–1 cropping season (**Table 2**).

Inoculation with PGPBs and foliar nano-Zn application positively increased the number of rows per cob of maize (**Table 2**). The maximum number of rows cob^−1^ were observed in plants that had been inoculated with *B. subtilis* and *P. fluorescens* in the 2019–20 and 2020–1 cropping seasons, respectively. These results were statistically similar to other inoculation treatments in relation to the control. In addition, foliar nano-Zn application increased the number of rows cob^−1^ of maize in the second cropping season only. The interactions of inoculation with PGPBs and foliar nano-Zn application for number of rows cob^−1^ were not significant in both years of study (**Table 2**).

In addition, the number of grains cob^−1^ of maize was significantly influenced by inoculation with PGPBs and foliar nano-Zn application, whereas their interactions were not significant in the 2019–20 and 2020–1 cropping seasons (**Table 3**). The number of grains cob^−1^ was increased by 11.9% and 15% with the inoculation of *B. subtilis* and *P. fluorescens* in the first and second maize cropping seasons, respectively, in comparison to those maize crops without inoculation treatments. The foliar nano-Zn application increased the number of grains cob^−1^ by 10.4% and 16.6%, compared with the control (i.e., absence of foliar Zn spray).

**Table 3:** Number of grains cob^-1^, 100-grains weight, and grain yield as a function of plant growth-promoting bacteria inoculation, together with or without nano-zinc oxide spray in the 2019–20 and 2020–1 cropping seasons.

Treatment	Number of grains cob^-1^			100 grains weight		Grain yield	
	—————			——– g ——–		—— kg ha^-1^ ——	
	2019–20	2020–1		2019–20	2020–1	2019–20	2020–1
Inoculation (I)							
Without	614 b		557 b	30.6 b	27.5 b	7172	7241
*A. brasilense*	675 a		623 a	33.5 a	30.1 a	8015	8521
*B. subtilis*	687 a		560 ab	32.7 ab	29.7 a	8405	8447
*P. fluorescens*	654 ab		642 a	31.8 ab	30.0 a	7866	8693
Foliar zinc (ZnF) spray (kg ha^-1^)							
0	625 b		559 b	31.3 b	28.5 b	7620	7730
3	690 a		652 a	32.9 a	30.2 a	8109	8720
F-test							
I	8.1^**^		11.3^**^	4.4^*^	10.5^**^	20.1^**^	19.9^**^
ZnF	32.7^**^		72.8^**^	7.9^*^	19.4^**^	18.1^**^	44.2^**^
I x ZnF	0.5^ns^		1.8^ns^	0.12^ns^	1.9^ns^	3.3^*^	6.1^**^
**CV (%)**	4.9		5.1	5.2	3.6	4.1	5.1

Means in the column followed by different letters are statistically different by Tukey’s test, p ≤ 0.05. ^**^ and ^*^—significant at p< 0.01 and p< 0.05, respectively, while ^ns^ is non-significant by F-test.

The interaction of inoculation with PGPBs and foliar nano-Zn application was not significant for 100-grains weight of maize (**Table 3**). Inoculation with *A. brasilense* increased 100-grains weight of maize by 9.47% and 9.45% in the 2019–20 and 2020–1 cropping seasons, respectively, which was statistically similar to the inoculation of *B. subtilis* and *P. fluorescens* compared with the without inoculation treatments. Foliar application of nano-Zn at the dose of 3 kg ha^−1^ increased 100-grains weight by 5.1% and 5.9% in the 2019–20 and 2020–1 maize cropping seasons, respectively (**Table 3**).

The effect of inoculation with PGPBs and foliar nano-Zn application, and their interactions were significant for maize grain yield in the 2019–20 and 2020–1 growing seasons (**Table 3**). The treatment with inoculations of *B. subtilis* and *P. fluorescens* increased maize grain yield by 17.2% and 20.1% in the 2019–20 and 2020–1 cropping seasons, respectively, in relation to the without inoculation treatments. In addition, foliar-applied nano-Zn also increased grain yield of maize by 6.4% and 12.8% in comparison to the control treatments. In case of interactions, the treatments with inoculation of *B. subtilis* performed better with nano-Zn foliar spray in the first maize cropping season (**Figure 3B**). In addition, maize plants inoculated with *P. fluorescens* had a greater grain yield in the presence of nano-Zn foliar application, which was statistically at per with treatments inoculated with *B. subtilis* and foliar nano-Zn application in the 2020–1 cropping season (**Figure 3C**). In general, the treatments inoculated with PGPBs produced greater grain yields regardless of foliar nano-Zn application in both cropping seasons. The lowest grain yield of maize was noted in the control treatments in both cropping seasons (**Figures 3B, C**).

### 3.2 Shoot and grain zinc accumulation and use efficiencies

There was positive influence of the treatments on shoot Zn accumulation of maize; however, their interactions were not significant in the 2019–20 and 2020–1 cropping seasons (**Table 4**). Inoculation with *P. fluorescens* improved shoot Zn accumulation by 30% and 51% in first and second maize cropping seasons, respectively, compared with the without inoculation treatments. In addition, foliar nano-Zn application improved shoot Zn accumulation by 35% and 36% in first and second cropping seasons, respectively, in comparison with the control treatments.

**Table 4:** Shoot zinc accumulation (SZnA) and grain zinc accumulation (GZnA), zinc use efficiency (ZnUE), and applied zinc recovery (AZnR) as a function of plant growth-promoting bacteria inoculation, together with or without nano-zinc oxide spray in the 2019–20 and 2020–1 cropping seasons.

Treatment	SZnA		GZnA		ZnUE		AZnR	
	———— g ha^-1^ ————				—– kg kg^-1^ —–		—— % ——	
	2019–20	2020–1	2019–20	2020–1	2019–20	2020–1	2019–20	2020–1
Inoculation (I)								
Without	333 b	306 b	216	211	357 c	321 b	76	53 b
*A. brasilense*	330 b	379 ab	288	287	619 b	676 ab	95	92 ab
*B. subtilis*	379 ab	372 ab	322	287	876 a	816 a	147	111 ab
*P. fluorescens*	433 a	462 a	285	318	535 b	1016 a	136	160 a
Foliar zinc (ZnF) spray (kg ha^-1^)								
0	314 b	322 b	236	243	—	—	—	—
3	423 a	438 a	320	309	—	—	—	—
F-test								
I	3.7^*^	6.23^**^	28^**^	13.6^**^	78.3^**^	11.1^**^	3.9^ns^	7.1^*^
ZnF	18.9^**^	20.7^**^	102^**^	28.0^**^	—	—	—	—
I x ZnF	0.5^ns^	0.37^ns^	10.8^*^	4.3^*^	—	—	—	—
**CV (%)**	19.2	19.0	8.5	12.7	8.2	24.9	30.2	32.2

Means in the column followed by different letters are statistically different by Tukey’s test, p ≤ 0.05. ^**^ and ^*^significant at p< 0.01 and p< 0.05, respectively, while ^ns^ is non-significant by F-test.

**Figure 4:**
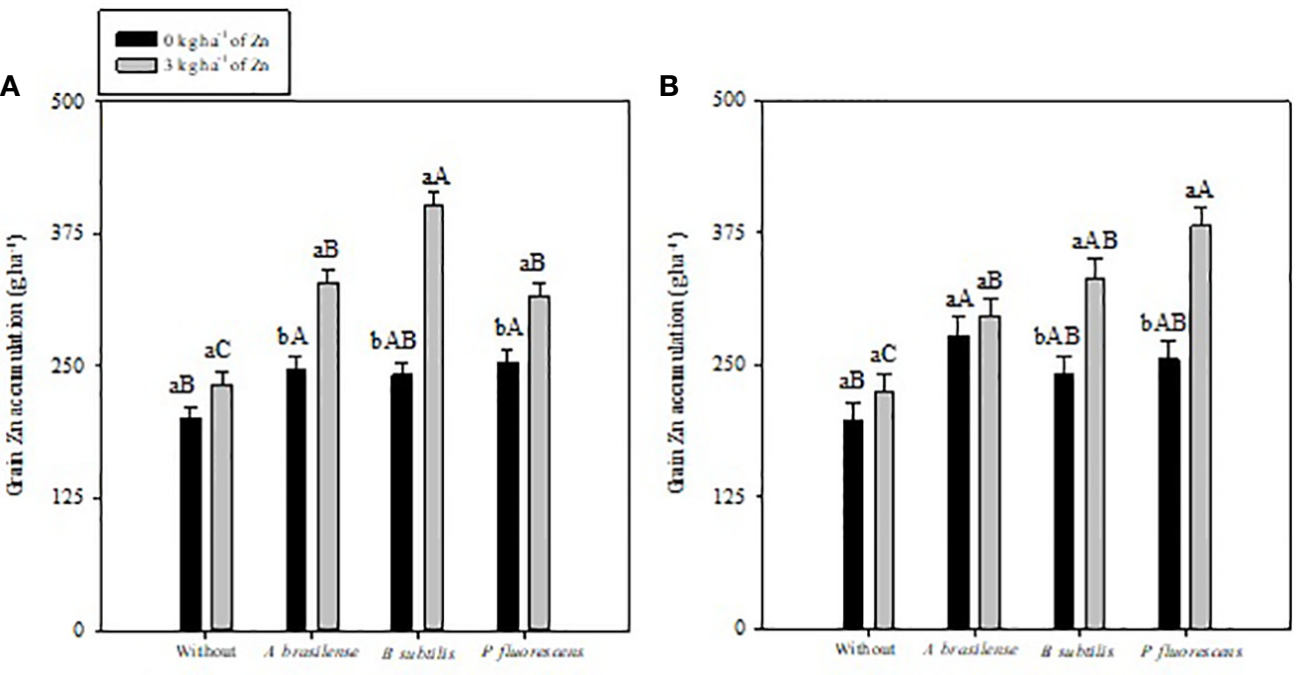
Maize grain zinc (Zn) accumulation in 2019–20 **(A)** and 2020–1 **(B)** as a function of plant growth-promoting bacteria with or without foliar zinc oxide (ZnO) application. Without = control (no inoculation). The uppercase letters compare interactions of inoculations within each dose of foliar nano-ZnO application and lowercase letters are used to compare interactions of foliar Zn doses (presence and absence) within each inoculation treatment. The identical alphabetic letters do not differ from each other by Tukey’s test (*p*< 0.05) for foliar ZnO doses and inoculations in the 2019–20 and 2020–1 cropping seasons. Error bars indicate standard error of the mean (*n* = 4 replications).

Inoculation with PGPBs and foliar nano-Zn application had a positive influence on grain Zn accumulation of maize in the 2019–20 and 2020–1 cropping seasons (**Table 4**). The interactions of PGPBs and foliar nano-Zn application for grain Zn accumulation were also significant (**Figure 4A**). Inoculation with *B. subtilis* and *P. fluorescens* in combination with foliar nano-Zn application improved grain Zn accumulation by 49% and 51% in the first and second maize cropping seasons, respectively (**Figures 4A, B**). The treatments with inoculation of *P. fluorescens* and *A. brasilense* performed better regardless of foliar nano-Zn application in both cropping seasons. In addition, the lowest grain Zn accumulation was observed in the treatments without inoculation of PGPBs and nano-Zn application in both maize cropping seasons (**Figures 4A, B**).

ZnUE and AZnR were increased in the treatments with inoculation of PGPBs and foliar nano-Zn application (**Table 4**). The treatments with inoculation of *B. subtilis* increased ZnUE by 145% in the 2019–20 cropping season. Interestingly, inoculation with *P. fluorescence* increased ZnUE by 216% in the second season, which was statistically at per with the treatments of inoculation with *B. subtilis* and *A. brasilense*, compared with the without inoculation treatment (**Table 4**). In addition, the treatments with inoculation of PGPBs positively influenced AZnR in the second maize cropping season only (**Table 4**). Inoculation with *P. fluorescence* was observed with a higher AZnR (160%), which was statistically similar to the treatment with inoculation of *B. subtilis* (111%) and *A. brasilense* (92%), than the without inoculation in the 2020–1 cropping season (**Table 4**).

### 3.3 Photosynthetic pigments

**Figure 5:**
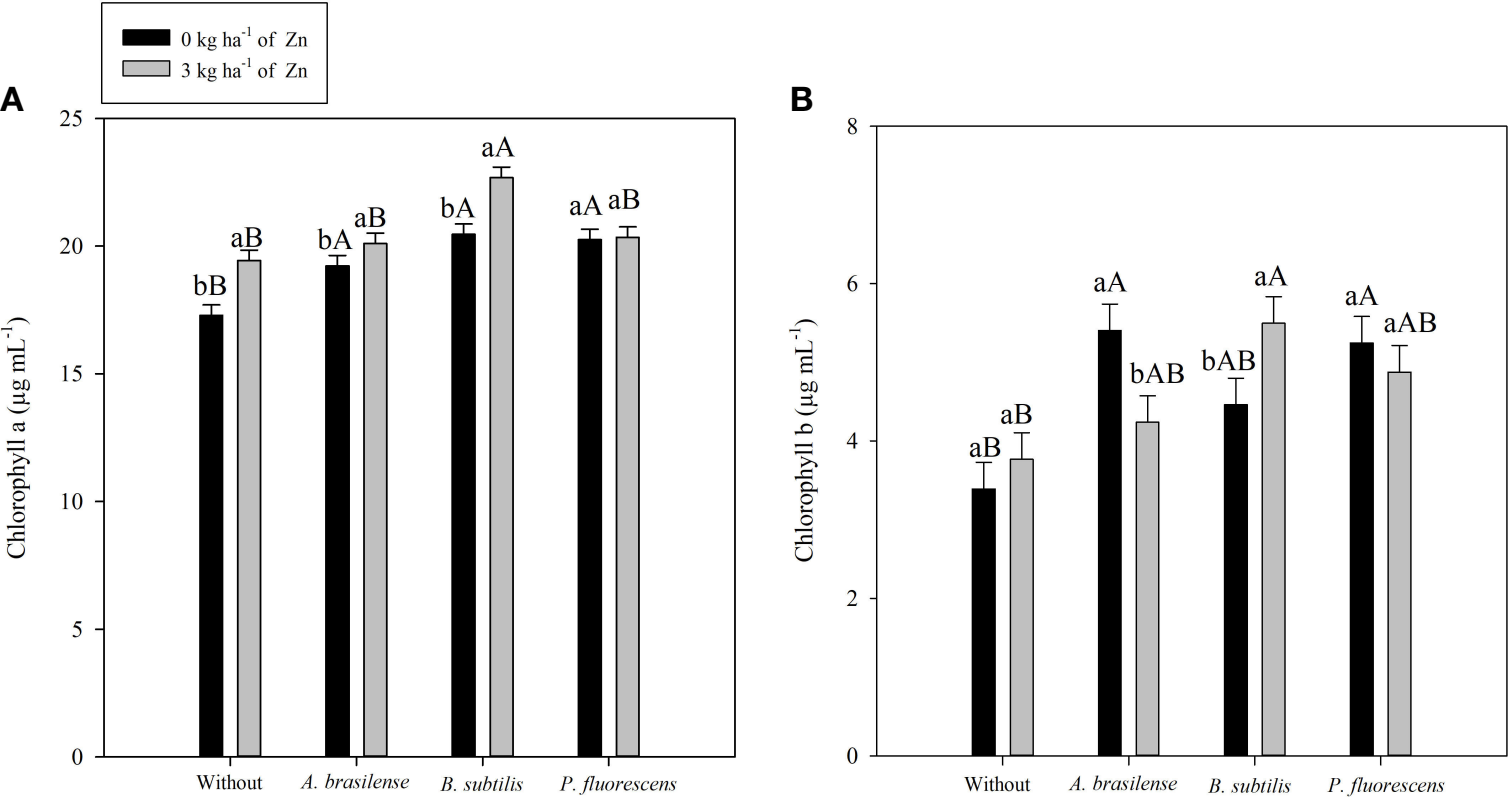
Concentrations of chlorophyll a in 2020–1 **(A)** and chlorophyll b in 2019–20 **(B)** of maize leaf as a function of plant growth-promoting bacteria with or without foliar zinc oxide (ZnO) application. Without = control (no inoculation). The uppercase letters compare interactions of inoculations within each dose of foliar nano-ZnO application and the lowercase letters are used to compare interactions of foliar zinc doses (presence and absence) within each inoculation treatment. The identical alphabetic letters do not differ from each other by Tukey’s test (*p*< 0.05) for foliar ZnO doses and inoculations in the 2019–20 and 2020–1 cropping seasons. Error bars indicate standard error of the mean (*n* = 4 replications).

There was positive impact of PGPB inoculation and foliar nano-Zn spray on the photosynthetic pigments of maize leaves at the flowering stage (**Table 5**). The interaction and seed inoculation with PGPBs did not affect chlorophyll a content in the 2019–20 maize cropping season. However, the effect of treatments and their interaction for chlorophyll a content in maize leaves was significant in the 2020–1 cropping season (**Table 5**; **Figure 5A**). Inoculation with *B. subtilis* and foliar nano-Zn spray was observed with highest chlorophyll a content, compared with other inoculation and without inoculation treatments (**Figure 5A**). In addition, the treatments with inoculation of *P. fluorescence* were observed with a higher chlorophyll a content in the absence of foliar nano-Zn application than with other treatments (**Figure 5A**). The lowest leaf chlorophyll a content was observed in control treatments (**Figure 5A**). Foliar nano-Zn spray at the dose of 3 kg ha^-1^ increased chlorophyll a content by 5.7% and 6.7% in the first and second cropping seasons, respectively, in comparison to the without nano-Zn foliar spray (**Table 5**).

**Table 5:** Photosynthetic pigment of maize leaves as a function of plant growth-promoting bacteria inoculation, together with or without nano-zinc oxide spray, in the 2019–20 and 2020–1 cropping seasons.

Treatment	Chlorophyll a		Chlorophyll b		Total chlorophyll		Carotenoids	
	———————————— μg mL^-1^ ————————————							
	2019–20	2020–1	2019–20	2020–1	2019–20	2020–1	2019–20	2020–1
Inoculation (I)								
Without	19.3	18.4	3.57	2.99 b	22.6 b	23.7 b	2.15 a	1.78 b
*A. brasilense*	20.4	19.7	4.82	4.47 a	24.8 ab	26.6 a	2.48 a	2.69 a
*B. subtilis*	20.1	21.6	4.97	4.8 a	26.0 a	27.7 a	2.61 a	3.25 a
*P. fluorescens*	19.9	20.3	5.10	3.98 ab	24.9 ab	26.2 a	2.68 a	2.85 a
Foliar zinc (ZnF) spray (kg ha^-1^)								
0	19.4 b	19.3	4.6	3.34 b	22.3 b	24.7 b	2.29 b	2.12 b
3	20.5 a	20.6	4.5	4.78 a	26.8 a	27.4 a	2.67 a	3.17 a
F-test								
I	2.3^ns^	21.4^**^	8.4^**^	6.6^**^	3.8^*^	7.8^**^	1.7^ns^	11.8^**^
ZnF	14.9^*^	21.4^**^	0.02^ns^	22.3^**^	38.1^**^	19^**^	4.36^*^	33.6^**^
I x ZnF	0.3^ns^	3.2^*^	3.9^*^	0.34^ns^	0.31^ns^	1.3^ns^	0.47^ns^	0.65^ns^
**CV (%)**	4.1	4.1	14.7	21.2	8.5	6.6	20.9	19.4

Means in the column followed by different letters are statistically different by Tukey’s test, p ≤ 0.05. ^**^ and ^*^ significant at p< 0.01 and p< 0.05, respectively, while ^ns^ is non-significant by F-test.

The interaction of PGPB inoculation and foliar nano-Zn spray for chlorophyll b was significant in the 2019–20 maize cropping season, whereas it was not significant in the 2020–1 maize cropping season (**Table 5**). Inoculation with *B. subtilis* in combination with foliar nano-Zn foliar spray was observed with highest chlorophyll b content, which was statistically similar to the inoculation of *A. brasilense* and foliar nano-Zn spray in the first maize cropping season (**Figure 5B**). The lowest chlorophyll b content was noted in the treatments without inoculation and foliar nano-Zn spray (**Figure 5B**). In addition, foliar nano-Zn spray did not influence leaf chlorophyll b content in the first season. Interestingly, foliar nano-Zn spray increased leaf chlorophyll b content by 43% in the 2020–1 cropping season, compared with the control treatment (**Table 5**).

The interactions of PGPBs and foliar nano-Zn spray for total chlorophyll content were not significant in both cropping seasons studied (**Table 5**), although, leaf total chlorophyll content was positively influenced by the treatment effects. Seeds inoculation with *B. subtilis* increased total chlorophyll content by 15% and 16.8% in the 2019–20 and 2020–1 cropping seasons, respectively, which was statistically similar to the inoculation treatments with *P. fluorescens* and *A. brasilense*, when compared with the without inoculation treatments (**Table 5**). In addition, foliar nano-Zn spray at the dose of 3 kg ha^-1^ increased total chlorophyll content of maize leaves by 20.2% and 10.9% in the first and second cropping seasons, respectively.

Leaf carotenoids content of maize was only significantly influenced by inoculation treatments in the 2020–1 cropping season, while foliar nano-Zn was observed to have a positive impact on carotenoid content in both cropping seasons (**Table 5**). Inoculation with *B. subtilis* increased leaf carotenoids content by 82.6%, which was statistically at per with other inoculations treatments during the second maize cropping season when compared with the without inoculation treatments (**Table 5**). In addition, the treatment with foliar nano-Zn spray increased leaf carotenoids content by 16.6% and 49.5% in the first and second cropping seasons, respectively, as compared with the control (**Table 5**).

### 3.4 Total soluble sugar, amino acids, and storage proteins

**Figure 6:**
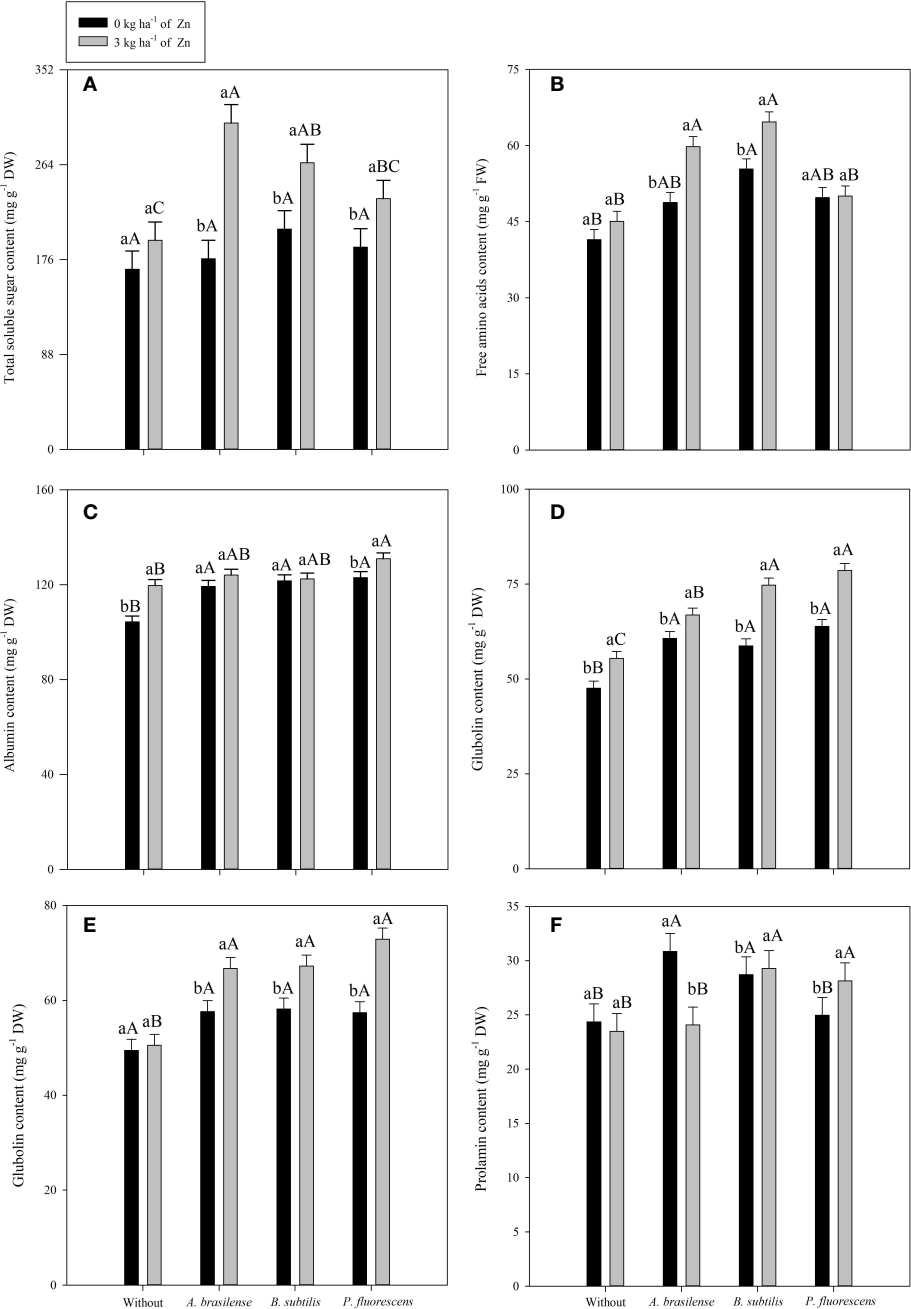
Concentrations of total soluble sugar in 2019–20 **(A)**, free amino acids in 2020–1 **(B)**, albumin in 2020–1 **(C)**, globulin in 2019 and 2020 (**D, E** respectively) and prolamin in 2020–1 **(F)** as a function of plant growth-promoting bacteria with or without foliar zinc oxide (ZnO) application. Without = control (no inoculation). The uppercase letters compare interactions of inoculations within each dose of foliar nano-ZnO application and the lowercase letters are used to compare interactions of foliar zinc doses (presence and absence) within each inoculation treatment. The identical alphabetic letters do not different from each other by Tukey’s test (*p*< 0.05) for foliar ZnO doses and inoculations in the 2019–20 and 2020–1 cropping seasons. Error bars indicate standard error of the mean (*n* = 4 replications).

TSS content in maize leaves was significantly influenced by the inoculation treatments and the foliar nano-Zn spray in both cropping seasons (**Table 6**). Inoculation with *A. brasilense* increased TSS content in leaves by 33% and 40% in the 2019–20 and 2020–1 cropping seasons, respectively, when compared with the without inoculation treatments (**Table 6**). In addition, foliar nano-Zn spray increased TSS content by 35% and 56% in the first and second cropping seasons of maize, respectively, compared with the control. The interaction of inoculation and foliar nano-Zn spray was significant in the 2019–20 cropping season only (**Table 6**). The treatments with co-application of *A. brasilense* and foliar nano-Zn spray at a dose of 3 kg ha^-1^ were observed with the highest total soluble sugar content in maize leaves (**Figure 6A**). The treatments with foliar nano-Zn application and without PGPB inoculation were observed with the lowest TSS content in leaves of maize. However, treatments without foliar nano-Zn application and inoculation with *B. subtilis* were observed with higher TSS content, which was statistically at per with other inoculation treatments (**Figure 6A**).

**Table 6:** Total soluble sugar (TSS), free amino acids, and albumin concentration as a function of plant growth-promoting bacteria inoculation, together with or without nano-zinc oxide spray, in the 2019–20 and 2020–1 cropping seasons.

Treatment	TSS		Free amino acids		Albumin	
	———————————— mg g^-1^ DW ————————————					
	2019–20	2020–1	2019–20	2020–1	2019–20	2020–1
Inoculation (I)						
Without	180	173 b	42.3 c	43.2	109 c	112
*A. brasilense*	240	242 a	51.2 b	54.3	119 bc	122
*B. subtilis*	235	224 ab	58.5 a	60.0	125 ab	122
*P. fluorescens*	210	204 ab	51.9 b	49.9	131 a	127
Foliar zinc (ZnF) spray (kg ha^-1^)						
0	184	165 b	47.8 b	48.8	116 b	117
3	249	257 a	54.1 a	54.9	126 a	124
F-test						
I	5.2^*^	4.15^*^	24.6^**^	25.5^**^	12.7^**^	12.8^**^
ZnF	29.1^**^	39.7^**^	22.6^**^	18.6^**^	12.8^*^	16.8^**^
I x ZnF	3.2^*^	1.8^ns^	1.12^ns^	3.12^*^	0.19^ns^	3.1^*^
**CV (%)**	15.7	19.5	7.4	7.6	6.0	4.1

Means in the column followed by different letters are statistically different by Tukey’s test, p ≤ 0.05. ^**^ and ^*^ significant at p< 0.01 and p< 0.05, respectively, while ^ns^ is non-significant by F-test.

The content of free amino acids in maize leaves was positively influenced by inoculation treatment and foliar nano-Zn spray in the 2019–20 and 2020–1 cropping seasons. The interaction was significant in only the second cropping season of maize (**Table 6**). Inoculation with *B. subtilis* increased amino acids content by 38.3% and 38.9% in the first and second cropping seasons, respectively, compared with the without inoculation treatment. Foliar nano-Zn spray also enhanced free amino acids content by 13.2% and 12.5% in the 2019–20 and 2020–1 maize cropping seasons, respectively, in comparison to the control. The interaction demonstrated that the treatments with foliar Zn spray at a dose of 3 kg ha^-1^ under inoculation of *B. subtilis* and *A. brasilense* increased the free amino acid content in maize leaves, compared with the without inoculation treatment (**Figure 6B**). Among PGPB inoculations, the treatments with *B. subtilis* were observed to have the higher amino acid content in the absence of foliar nano-Zn application. The lowest amino acid content was observed in the control treatments (**Figure 6B**).

Grain storage proteins of maize were significantly influenced by inoculation with PGPBs and nano-Zn foliar spray (**Tables 6**, **7**). Inoculation with *P. fluorescens* enhanced grain albumin concentration by 20.2% and 13.4% in the first and second cropping seasons, respectively, as compared with the without inoculation treatments (**Table 6**). Foliar nano-Zn spray also improved grains albumin concentration by 8.6% and 5.9% in the first and second cropping seasons, respectively, as compared with the control treatments. The interaction was significant in only the second cropping season, in which the highest grain albumin concentration was observed with the combined application of *P. fluorescens* inoculation and foliar nano-Zn spray, compared with the rest of the treatments (**Figure 6C**). All treatments with inoculation of PGPBs had improved grain albumin concentration regardless of the foliar nano-Zn application. The lowest albumin concentration was observed in the treatments without inoculation and nano-Zn application (**Figure 6C**).

**Table 7:** Globulin, glutelin and prolamin concentration of maize grains as a function of plant growth-promoting bacteria inoculation, together with or without nano-zinc oxide spray, in the 2019–20 and 2020–1 cropping seasons.

Treatment	Globulin		Glutelin		Prolamin	
	———————————— mg g^-1^ DW ————————————					
	2019–20	2020–1	2019–20	2020–1	2019–20	2020–1
Inoculation (I)						
Without	51	50	190 b	205 b	24.8 a	23.9
*A. brasilense*	64	62	215 a	222 a	26.5 a	27.5
*B. subtilis*	67	63	219 a	223 a	28.5 a	28.9
*P. fluorescens*	71	65	216 a	219 a	25.1 a	26.5
Foliar zinc (ZnF) spray (kg ha^-1^)						
0	58	56	203 b	214 b	24.2 b	26.2
3	69	64	216 a	220 a	28.2 a	27.2
F-test						
I	41.8^**^	17.3^**^	15.6^**^	14.2^**^	1.4^ns^	3.3^*^
ZnF	73.4^**^	28.3^**^	16.2^**^	7.0^*^	8.5^*^	0.71^ns^
I x ZnF	3.6^*^	3.27^*^	0.12^ns^	0.52^ns^	0.55^ns^	3.3^*^
**CV (%)**	5.8	7.7	4.5	2.9	15	12.3

Means in the column followed by different letters are statistically different by Tukey’s test, p ≤ 0.05. ^**^ and ^*^ significant at p< 0.01 and p< 0.05, respectively, while ^ns^ is non-significant by F-test.

The interactive effect of inoculation × foliar nano-Zn spray was significant for grain globulin concentration in both cropping seasons (**Table 7**, **Figures 6D, E**). The highest grain globulin concentration was observed with foliar nano-Zn fertilization under inoculation with *P. fluorescens*, which was statistically similar to the with inoculation of *B. subtilis* treatment in the 2019–20 (**Figure 6D**), and the with *B. subtilis* and *A. brasilense* in 2020–1 (**Figure 6E**) maize cropping seasons. The treatments with inoculation of PGPBs were observed to have higher globulin concentrations in the maize grains, even in the absence of foliar nano-Zn application, than the without inoculation treatments. The lowest globulin concentration in both studies was noted in the treatments without inoculation and nano-Zn application (**Figures 6D, E**).

The interaction of inoculations × foliar nano-Zn for glutelin concentration was not significant in both cropping seasons studied (**Table 7**). Grain glutelin concentration was improved by 15.3% and 8.8% with inoculation of *B. subtilis* in the first and second cropping seasons, respectively, which was statistically at per with other inoculation, when compared with the without inoculation treatments. Foliar nano-Zn spray improved grain glutelin concentration by 6.4% and 2.8% in the 2019–20 and 2020–1 cropping seasons in comparison to the control (**Table 7**).

Grain prolamin concentration of maize was not significantly influenced by inoculation and interaction of inoculation × nano-Zn spray in the 2019–20 cropping season, while the effect of only foliar nano-Zn spray was not significant in the 2020–1cropping season (**Table 7**). Foliar nano-Zn spray improved grain prolamin concentration of maize by 16.5% in the second cropping season. In the 2020–1 cropping season, inoculation with *B. subtilis* and *P. fluorescens* along with foliar nano-Zn spray were observed to have higher prolamin concentrations in the second cropping season (**Figure 6F**). In addition, inoculation with *A. brasilense* was observed with the highest grain prolamin concentration in the absence of foliar nano-Zn spray application. The treatments without inoculation were observed with the lowest grain prolamin concentration, regardless of the nano-Zn application (**Figure 6F**).

### 3.5 Pearson’s correlation among evaluated attributes of maize

**Figure 7:**
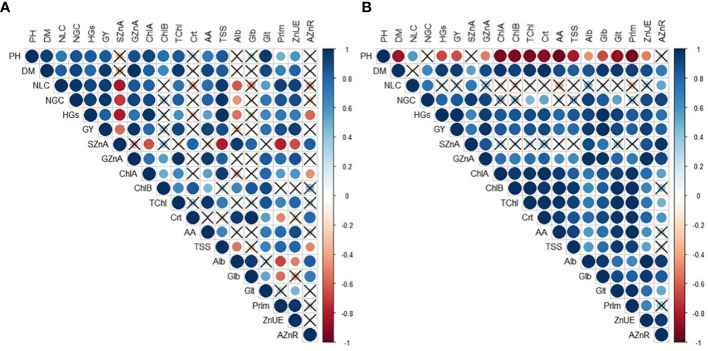
Heatmap color scale indicating Pearson’s correlation among evaluated attributes of maize in response to plant growth-promoting bacteria and foliar nano-zinc oxide applications in the 2019–20 **(A)** and 2020–1 **(B)** cropping seasons. "×" indicates a non-significant relationship (*p* ≤ 0.05). PH, plant height; DM, shoot dry matter; NLC, number of row per cob; NGC, number of grains per cob; HGs, 100-grains weight; GY, grain yield; SZnA, shoot Zn accumulation; GZnA, grain Zn accumulation; ChlA, chlorophyll a; ChlB, chlorophyll b; TChl , total chlorophyll; Crt, carotenoids; AA, free amino acids; TSS, total soluble sugar; Alb, albumin; Glb, globulin; Glt, glutelin; Prlm , prolamin; ZnUE, Zn use efficiency; and AZnR, applied Zn recovery.

There were positive and significant correlations between Zn use efficiency and plant height, shoot dry matter, yield components, grain yield, grain Zn accumulation, chlorophyll a, amino acids, TSS, glutelin, and prolamin concentration of maize, regardless of the treatments applied in the 2019–20 cropping season (**Figures 7A**). A positive correlation was observed between Zn use efficiency and shoot and grain Zn accumulation, applied Zn recovery, shoot dry matter, grain yield, and photosynthetic and biochemical attributes of maize in the 2020–1 cropping season (**Figure 7B**).

In addition, there were positive and significant correlations between grain yield and Zn use efficiency, shoot dry matter, yield components, grain Zn accumulation, chlorophyll a, amino acids, total chlorophyll, and prolamin concentration of maize, regardless of the treatments applied in the 2019–20 crop season (**Figures 7A**). A positive correlation was observed between grain yield and all growth and yield components, as well as photosynthetic and biochemical attributes of maize in the 2020–1 crop season (**Figure 7B**).

### 3.6 Principal component analysis among evaluated attributes of maize

**Figure 8:**
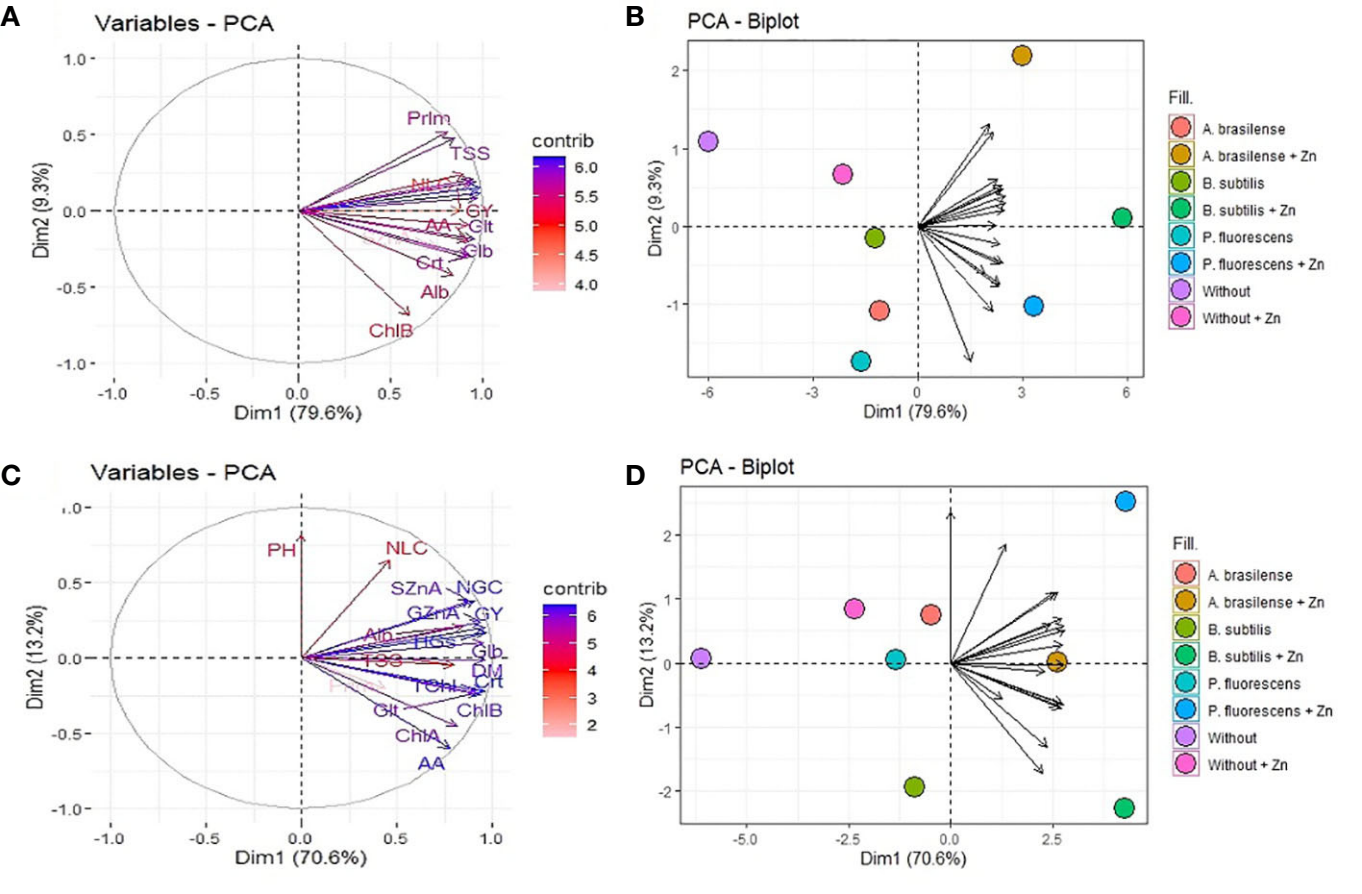
Loadings and biplot graphics of principal component analysis among the relationship of plant growth-promoting bacteria and foliar nano-zinc oxide applications for growth, nutritional, yield, and biochemical attributes of maize in the 2019–20 **(A, B)** and 2020–1 **(C, D)** cropping seasons. PH, plant height; DM, shoot dry matter; NLC, number of row per cob; NGC, number of grains per cob; HGs, 100-grains weight; GY, grain yield; SZnA, shoot Zn accumulation; GZnA, grain Zn accumulation; ChlA, chlorophyll a; ChlB, chlorophyll b; TChl, total chlorophyll; Crt, carotenoids; AA, free amino acids; TSS, total soluble sugar; Alb, albumin; Glb, globulin; Glt, glutelin; and Prlm, prolamin.

PCA was performed to investigate the changes in the yield, nutritional, and biochemical attributes of maize in the 2019–20 and 2020–1 cropping seasons (**Figure 8**). The eigenvalues of all eight principal components were greater than 1 and account for 100% of the data variation in both maize cropping seasons (**Supplementary Tables 1**, **2**). The PC1 explained 79.6% and 70.6% of the data cumulative variation, while PC2 represented 88.9% and 82.7% in the 2019–20 and 2020–1 cropping seasons, respectively. The biplot graphs of PC1 and PC2 indicated that the group formed by inoculation with *A. brasilense, B. subtilis*, and *P. fluorescens* with foliar nano-Zn spray at a dose of 3 kg ha^-1^ obtained a positive correlation for all analyzed maize parameters in the first cropping season (**Figures 8A, B**). While analyzing the biplot graph of grouped PC1 and PC2 in the 2020–1 cropping season, all analyzed parameters showed a positive correlation with the group formed by inoculation with PGPBs, except plant height (**Figures 8C, D**).

## 4 Discussion

Plants’ adaptation and responses to nutrients deficiency are being satisfied by ensuring minimal requirements and a carbon trade-off cost. Several strategies are being adapted to protect plants from the damages of harsh environmental conditions, especially in tropical rain-fed regions (Galindo et al., 2021). In this scenario, limited literature is available on the use of PGPBs in combination with foliar nano-ZnO on the growth and performance of maize. To address this incessant problem, the current study used Zn-improving PGPBs, such as *A. brasilense*, *B. subtilis*, and *P. fluorescens* in combination with a foliar nano-Zn application to assist biochemical attributes, primary metabolisms, and yield of maize crop in the tropical savannah.

The positive correlation between maize growth and yield, shoot–grain accumulation, photosynthetic pigments, and primary metabolism endorsed the hypothesis of the current study (**Figure 7**). Therefore, the current study is possible due to the synergetic effect of PGPBs with Zn enrichment and their role in different metabolic processes, nutrient use and acquisition, and synthesis of phyto-hormones (Mitter et al., 2017; Kudoyarova et al., 2019; Housh et al., 2021). In this context, the current study indicated that inoculation with *B. subtilis* and *P. fluorescence* along with foliar nano-Zn application produced taller plants (**Figure 3A**), greater shoot dry matter, higher number of rows, grains cobs^−1^, heavier 100-grains weight (**Tables 2**, **3**), and grain yield (**Figures 3B, C**) in two maize cropping seasons. It might be due to the role of PGPBs in nutrient solubilization and phytohormone production that stimulates nutrient availability and absorption through the roots, as well the role Zn plays in cell multiplication and protein synthesis (Rossi et al., 2019; Swarnalakshmi et al., 2020). The individual or combined use of PGPBs with Zn could modulate phosphatase and invertase activities in soil as well as proline activities in plants (Tanveer et al., 2022), thus contributing to higher levels of plant growth and yield as an end product (Jalal et al., 2022a; Jalal et al., 2022b). It has also reported that inoculation with PGPBs promotes maize growth by regulating phytohormones and growth regulators, which could improve nutrient solubilization and nutrient uptake for better plant growth and production (Oleńska et al., 2020; Ribeiro et al., 2022). It has been reported that PGPBs are associated with greater root–shoot biomass and dry matter, which can lead to the promotion of vegetative growth at an early stage and a greater productivity at maturity (Sousa et al., 2021; Jalal et al., 2022b). In addition, foliar spray of nano-fertilizer improves growth and yield of maize by enhancing plant biochemical processes and resistance against ROS (Babaei et al., 2017). Zn regulates different biochemical attributes of plants through cell elongation and multiplication (as a result of auxin synthesis), thus leading to a greater biomass and productivity of cereal crops (Doolette et al., 2020; Jalal et al., 2022c). Despite of all this, the non-significant effect of foliar nano-Zn application for plant height and number of row cobs^−1^ in the first and second maize cropping season (**Table 2**) may be because plant nutrition with Zn was adequate, while these parameters are more influenced by genetic factors and tropical climatic conditions (**Figure 2**). In addition, low foliar Zn supply is another factor that can cause physiological and leaf anatomical alterations that can, consequently, affect nutrient penetration and accumulation, depending on the deficiency of target nutrient (Brian, 2008).

Results of the current study indicated that inoculation with PGPBs, such as *B. subtilis* and *P. fluorescens*, in combination with foliar nano-Zn application improved Zn accumulation in shoot tissue (**Table 4**) and grains (**Figures 4A, B**), as well as increased ZnUE and AZnR in maize crop cultivation (**Table 4**). It may be due to the positive interception of PGPBs in scavenging roots to produce growth-promoting hormones, and the increased water and nutrients uptake to shoot and grain tissues (Mumtaz et al., 2017). Previous studies indicated that applied inoculants interact with already existing microbes in the root rhizosphere, modifying root architecture, reducing phytic acid assimilation, and stimulating nutrient transportation to shoot and edible tissues (Singh and Prasanna, 2020). It has also reported that foliar Zn application could enhance translocation of Zn to shoot and edible tissues (Jalal et al., 2020; Jalal et al., 2022c) by developing coordination with amino acid (cysteine) and protein synthesis (Gupta et al., 2016). Foliar Zn application is an effective strategy to overcome the edaphic deficiency by improving bioavailability in edible tissue, leading to better biofortification (Mishra, 2022). In this context, the results of the present study are a progressive step to understanding the integrated use of PGPBs and foliar ZnO application for greater growth, yield, and biofortification of maize grains with higher Zn use efficiency (**Table 4**). It has been reported in another study that plant growth-promoting microbes are being identified as natural biofortifiers, synthesizing organic acids, acting as chelating agents, and producing siderophores that ultimately result in biofortification and higher crop yield in a sustainable manner (Ramesh et al., 2014; Upadhayay et al., 2022). Maize is one of the most important cereal crops for food and nutrition security, with high phytic acid and low Zn concentrations that may be the origin cause of malnutrition, especially in the regions under Zn deficiency like the tropical savannah of Brazil (Cakmak et al., 2010; Fageria et al., 2011). In this context, the present study exhibited that inoculation with *B. subtilis* and *P. fluorescens* along with foliar nano-Zn improved grain Zn accumulation (**Figure 4**) and Zn use efficiency in maize cultivation (**Table 4**). It has been reported that the inoculation with PGPBs in combination with foliar or soil Zn application contributes to the reduction of phytic acid, which consequently increases Zn concentration in the embryo, aleurone, endosperm, and whole grains of cereal crops (Rehman et al., 2018; Jalal et al., 2022b). In addition, foliar Zn spray considerably mobilized in the phloem compared with conventional soil Zn fertilization treatment, and the crop can deal with malnutrition because of its rapid remobilization and localization into the grains (Firdous et al., 2020; Rehman et al., 2021; Jalal et al., 2022c).

In the present study, the considerable increase in maize growth and Zn nutrition is due to the improvement of photosynthetic pigments (chlorophyll a and b, total chlorophyll, and carotenoids content) under inoculation with PGPBs and foliar nano-Zn application (**Table 5**). This increase in photosynthetic pigments might be due to the role of PGPBs in stabilizing the biochemical and physiological functions of plants, which can be attributable to stomatal conductance, transpiration, and intercellular gas exchange processes to increase photosynthetic rate of the plants (Pereira et al., 2020). The present results indicated that chlorophyll a, b, and total, and carotenoids concentrations were improved with the inoculation of *B. subtilis* and foliar Zn spray in both maize-cultivated seasons (**Table 5**, **Figures 5A, B**). It might be due to the critical role of PGPBs and Zn in the production of phytohormones, nitrogen fixation, and improving photosystem II efficiency (Seyed Sharifi et al., 2020). It has been reported that combined application of PGPBs and ZnO revealed itself as a promising technique to increase chlorophyll concentrations and performance of wheat (Azmat et al., 2022). The application of nano-Zn with PGPBs, including *Bacillus* and *Pseudomonas* sp., regulate defensive enzymes and intercellular homeostasis of plants to create optimal cellular conditions, which may lead to higher concentrations of photosynthetic pigments (Yasmin et al., 2020a). Batool et al. (2020) reported that inoculation with *B. subtilis* is an effective strategy that regulates chlorophyll a and b, and carotenoid content, as well as other biochemical process, thus leading to sustainable growth and production of the plants under harsh environmental conditions. In addition, the non-significant effect of inoculation with PGPBs on leaf concentrations of chlorophyll a and carotenoids (**Table 5**) might be because the plant nutrition with Zn was adequate and tropical climatic conditions (**Figure 2**). Despite this, inoculation of PGPBs attribute to competition of already existing microbial consortium in rhizophere, which can ultimately affect nutrient transportation and growth performance of maize (Tang et al., 2020).

There was a remarkable increase in the concentration of TSS, free amino acids, and grain storage proteins (albumin, globulin, glutalin, and prolamin) of maize when treated with PGPBs and foliar nano-Zn spray (**Tables 6**, **7**). It may be possible because of the role of foliar nano-Zn in upregulation of antioxidant systems and primary metabolites of the plant that contribute to enzyme activation and proteins synthesis (Ghani et al., 2022). Previous studies claimed that PGPBs regulate the production of photo-assimilates, interlinking the outcomes of foliar nano-Zn application with other physiological and biochemical functions that could ultimately improve primary metabolites in the leaves and storage proteins in grains of different crops (Batool et al., 2020; Azmat et al., 2022). The present results exhibited that combined application of PGPBs and foliar nano-Zn improved concentration of TSS (**Figure 6A**), free amino acids (**Figure 6B**), albumin (**Figure 6C**), globulin (**Figures 6D, E**), and prolamin (**Figure 6F**) in maize leaves and grains. The reason might be due to the rapid absorption and transportation of foliar nano-Zn with the involvement of several factors (i.e., thickness, density, and chemical composition of cuticle, trichomes, and stomata conductance), which are responsible for the operation of the entire plant machinery and, thus, improving metabolic and biochemical processes of the plants (Yumei et al., 2014; Xie et al., 2020). Zn fertilization increases grain reserve proteins because of its involvement in nitrate reductase activities and nitrogen assimilation pathways (Liu et al., 2015; Silva et al., 2021). Zn fertilization has also reported that co-application of Zn and PGPBs could modulate plant defensive system by improving photosynthetic pigments and primary metabolites, leading to better plant performance and yield (Tanveer et al., 2022). PGPBs induce multiple physiological functions by absorbing available nutrients through roots that may stimulate plant nutrition and primary metabolism in a sustainable manner (Yahaghi et al., 2019). The co-application of PGPBs and Zn improved TSS, amino acids, and protein content, leading to better performance and biofortification of maize (Upadhyay et al., 2021). Hence, inoculation with PGPBs and foliar nano-Zn application improved performance, primary metabolism, and the yield of maize. This strategy also proved to be a sustainable management practice for higher productivity and Zn biofortification of maize in tropical savannah conditions.

## 5 Conclusions

To satisfy the food and nutritious demands of an exponentially growing human population, use of PGPBs is one of the most sustainable and ecofriendly strategies that can increase nutrition, performance, productivity, and nutrient assimilation into the edible tissues of maize crop. In addition, foliar nano-Zn spray also proved to be a feasible and environmentally safe technique for improving Zn accumulation, growth, and biochemical attributes of maize. Therefore, it was verified from the current field findings that co-application of *B. subtilis* and nano-Zn at a dose of 3 kg Zn ha^−1^, applied in two splits, increased plant height, shoot dry matter, yield components, and yield of maize in tropical savannah. Zn accumulation in shoot and grains, as well as Zn use efficiency and applied Zn recovery, were also improved with inoculation of *B. subtilis* and *P. fluorescens*, along with foliar nano-Zn application. Chlorophyll a, b and total, carotenoids, TSS, free amino acids in the leaves, and storage proteins (albumin, globulin, glutelin, and prolamin) in grains of maize were improved with inoculation of *B. subtilis* in combination with foliar nano-Zn application. Therefore, seed inoculation with *B. subtilis* and *P. fluorescens* in combination with foliar nano-Zn application is considered to be a highly effective and low-cost alternative strategy to improve Zn acquisition and Zn use efficiency, biochemical and primary metabolism, with higher productivity of maize in tropical savannahs. The present study gives an insight on the interaction of PGPBs and foliar nano-Zn application about various morphological and biochemical aspects of maize. Using this information, prospective research should aim to know the molecular and laboratory mechanisms (translocation, localization, loading, transporter proteins, etc.) behind the higher accumulation and improved biochemical and physiological attributes of maize to better understand the responses of PGPBs in different edaphoclimatic conditions.

## Data availability statement

The raw data supporting the conclusions of this article will be made available by the authors, without undue reservation.

## Author contributions

Conceptualization, AJ and MT; methodology, AJ and CO; software, CO, GF, and FG; validation, AJ, FG; formal analysis, GF and AJ; investigation, AJ, GF and IG; resources; data curation, AJ, CO, AB, and PC; writing—original draft preparation, AJ; writing—review and editing, MT, EF, and FG; visualization, BL, PC, and IG; supervision, MT; project administration, AJ and MT; and funding acquisition, AJ and MT. All authors have read and agreed to the published version of the manuscript.
