# Supplementary material for: Nanozinc and plant growth-promoting bacteria improve biochemical and metabolic attributes of maize in tropical Cerrado
Source: Front Plant Sci. 2023 Jan 12;13:1046642. doi: 10.3389/fpls.2022.1046642 (PMC9878843; doi:10.3389/fpls.2022.1046642)
Supplement: Supplementary file 1 [file Table_1.docx]

**Journal: Frontiers in Plant Sciences**

Supplementary material

Nano-zinc and plant growth-promoting bacteria improve biochemical and metabolic attributes of maize in tropical Cerrado

Arshad Jalal^1^, Carlos Eduardo da Silva Oliveira^1^, Andréa de Castro Bastos^1^, Guilherme Carlos Fernandes^1^, Bruno Horschut de Lima^1^, Enes Furlani Junior^2^, Pedro Henrique Gomes de Carvalho^1^, Fernando Shintate Galindo^2^, Isabela Martins Bueno Gato^1^, Marcelo Carvalho Minhoto Teixeira Filho^1*^

^1^São Paulo State University (UNESP), Department of Plant Protection, Rural Engineering and Soils (DEFERS), Postal Code 15385-000, Ilha Solteira, SP, Brazil

^2^São Paulo State University (UNESP), Department of Plant Science, Food Technology and Socio-Economics, Postal Code 15385-000, Ilha Solteira, SP, Brazil

^3^Center for Nuclear Energy in Agriculture (CENA), University of São Paulo (USP), Piracicaba, Postal Code 13416-000, SP, Brazil

*** Correspondence:**Marcelo Carvalho Minhoto Teixeira Filho
mcm.teixeira-filho@unesp.br

**Sup Table 1.** Eigenvalue and percentage of variance and factor loadings generated by principal component analysis for maize in 2019-2020 cropping season.

**PC1 PC2 PC3 PC4 PC5 PC6 PC7 PC8**

Eigenvalues 1.43 1.67 1.09 3.67 2.56 1.93 8.71 2.93

Variability (%) 7.96 9.28 6.07 2.04 1.42 1.07 4.84 1.63

Cumulative variance (%) 79.6 88.90 94.97 97.02 98.44 99.5 100 100

**Sup Table 2.** Eigenvalue and percentage of variance and factor loadings generated by principal component analysis for maize in 2020-2021 cropping season.

**PC1 PC2 PC3 PC4 PC5 PC6 PC7 PC8**

Eigenvalues 1.27 2.37 1.36 7.75 4.60 2.35 8.82 5.84

Variability (%) 7.06 1.32 7.56 4.31 2.55 1.31 4.90 3.24

Cumulative variance (%) 70.62 83.78 91.34 95.64 98.20 99.51 100 100
